# Parent Attachment and Video Gaming Addiction: The Serial Mediation Role of Social Support and Maladaptive Daydreaming

**DOI:** 10.3390/ejihpe15040060

**Published:** 2025-04-13

**Authors:** Usman Ahmad Zaheer, Sofia Mastrokoukou, Claudio Longobardi, Paolo Bozzato

**Affiliations:** 1 Institute of Applied Psychology, University of the Punjab, Lahore 54782, Pakistan; usmanahmad.appsy@pu.edu.pk; 2 Department of Politica and Social Science, University of Salerno, 84084 Fisciano, Italy; smastrokoukou@unisa.it; 3 Department of Psychology, University of Turin, 10124 Torino, Italy; 4 Department of Human Sciences, Territory and Innovation, University of Insubria, 21100 Varese, Italy; paolo.bozzato@uninsubria.it

**Keywords:** video gaming addiction, adolescence, parent attachment, social support, maladaptive daydreaming

## Abstract

Previous research has demonstrated both direct and indirect relationships between parental attachment and gaming addiction in adolescents. This study aimed to investigate the role of parental attachment in adolescent gaming addiction, specifically examining how maladaptive daydreaming and perceived social support function as mediators in this relationship. A convenience sample of 898 Italian adolescents (550 female, *M*_age_ = 14.89 years, *SD* = 1.71) completed a questionnaire that included the Inventory of Parent and Peers Attachment, the Maladaptive Daydreaming Scale, the Game Addiction Scale, and a demographic survey. Correlation analyses confirmed a negative relationship between parental attachment and gaming addiction. Mediation analyses also showed that perceived social support and maladaptive daydreaming serve as significant serial mediators in this relationship. These results emphasize the importance of both factors in understanding how parental attachment influences gaming addiction. Adolescents exhibiting gaming addiction is caused by low perceived social support and maladaptive daydreaming may benefit from psychological interventions targeting adaptive regulation strategies. Strengthening the sense of security and self-confidence through such interventions may help to reduce excessive gaming behaviors.

## 1. Introduction

### 1.1. Video Gaming Addiction in Adolescence

The rapid growth of the video game industry has created increasingly immersive and social gaming experiences, which have been shown to foster problem-solving skills and social connections (Eichenbaum et al., 2014).

However, when gaming becomes excessive, it can have negative effects, such as poorer performance in school, increased impulsivity and aggression, and social disengagement (D. Kim et al., 2022). Adolescents are particularly at risk of becoming addicted to gambling due to their impulsivity and peer influence. Research suggests that developmental changes in adolescence promote increased engagement in addictive behavior (Yaacob et al., 2021).

The American Psychiatric Association (2022)’s DSM-5-TR and the World Health Organization (2019) have identified internet gaming disorder as symptoms that include: Preoccupation with gaming, withdrawal symptoms when gaming is discontinued, increased tolerance to the gaming stimulus, and continuation of gaming even when it negatively impacts a person’s life situation. In addition, the literature indicates that addictive gaming behavior in adolescents is associated with mental health problems such as anxiety and depression, increased social isolation, declining academic performance, and an overall deterioration in health as a result of physical inactivity (Männikkö et al., 2020). As advances in gaming technology continue to advance, identifying the psychological and social consequences associated with gaming addiction in adolescents will enable education and prevention approaches.

This paper aims to examine parental attachment and social support, maladaptive daydreaming, and adolescent gaming addiction to understand how and to what extent they may be associated with adolescent gaming addiction.

### 1.2. Parental Attachment and Video Gaming Addiction

Parental attachment is of fundamental importance for a person’s healthy psychological development. Attachment theory (Bowlby, 1982) posits that early parental responses form secure attachments, providing children with a sense of safety and emotional security to explore their environment. Insecure attachments, however, can lead to anxiety and maladaptive behaviors. However, there are also cases of insecure attachment, where inconsistent, neglectful, or overly intrusive caregiving disrupts this sense of security, and causes the child to develop anxiety, ambivalence, or avoidance in their relationship with their parents (Bowlby & Holmes, 2012).

This theory also states that children internalize the experiences they have with their parents and develop “internal working models” with significant caregivers that contribute to positive or negative development in adolescence. In fact, whether the attachment to a caregiver is secure or insecure influences the quality of future relationships. Healthy attachment is likely to maintain relationships with others in a positive manner, whereas unhealthy attachment can lead to difficulties in forming and maintaining healthy relationships with others (Rosenstein & Horowitz, 1996).

Even in adolescence, parent–child attachment remains important, alongside peer attachment, and plays an important role in mental health and self-esteem in adolescence (Wilkinson, 2004). Both parental attachment and peer attachment are considered secure attachments that positively influence identity development in adolescence (Meeus et al., 2002). On the other hand, insecure attachments, characterized by less trust, poor communication, and feelings of alienation, in combination with other factors can lead to internalizing symptoms, anxiety, and depression (Brumariu & Kerns, 2010), as well as externalizing behaviors such as delinquency (Brauer & De Coster, 2015), bullying and cyberbullying (Charalampous et al., 2018), internet use and abuse (Ballarotto et al., 2018).

The above evidence suggests that parental attachment is negatively associated with involvement in various types of gaming addiction. Indeed, Schneider et al. (2017), in their systematic review of familiar factors in problematic internet gaming, note that the most commonly studied familiar factor was the parent–child relationship. This factor is assessed, in most cases, with a self-reported measure of attachment. In a more recent systematic review by Nielsen et al. (2020), the authors conclude that protective factors, such as supportive parenting and healthy family dynamics, are associated with lower levels of problematic gaming, while risk factors, such as negative parenting and dysfunctional family interactions, are associated with higher levels. Overall, family and parental influences were as effective as the adolescents’ personal traits. These findings can be explained by Bronfenbrenner’s bioecological systems theory, which states that human development is shaped by the dynamic interplay between individuals and their multiple environmental systems, such as family, school, and community, all of which influence each other over time (Bronfenbrenner, 1986; Bronfenbrenner & Morris, 2007). In the context of gaming addiction and parental attachment, Bronfenbrenner’s theory helps to explain how family relationships, along with other individual and contextual factors, can influence adolescent behavior.

Some studies have shown that parental attachment is directly related to gaming addiction, while others have found an indirect relationship between these two variables. For example, K. Kim and Kim (2015) found a significant relationship between parental attachment and internet gaming addiction in a study of 624 middle and high school students in South Korea. In addition, Cui et al. (2018) found that a lack of parental attachment was a strong predictor of problematic gaming in 1553 Chinese adolescents. As expected, those who had a more intimate relationship with their parents were less likely to be addicted to gaming.

In contrast, some studies suggest that parental attachment has no direct influence on gaming addiction. Yun et al. (2012) showed that parental attachment was not significantly related to internet gaming addiction in middle school students. Throuvala et al. (2019) found that parental rejection had no direct influence on internet gaming disorder but was associated with it through the mediating effect of central self-evaluation. Similarly, Malik et al. (2020) reported that although parental attachment had no direct effect on gaming addiction, it did have an indirect effect via self-control. Teng et al. (2020) investigated the links between parent–adolescent attachment and internet gaming disorder and found that attachment to both the fathers and mother did not predict future internet gaming disorder. In the study, conducted by S. Kim and Chun (2022), no direct relationship was found between parental or peer attachment and gaming addiction. However, parental attachment significantly contributed to the reduction in gaming addiction via the mediating effect of social stigmatization in the studied group of out-of-school adolescents.

### 1.3. Social Support and Maladaptive Daydreaming as Serial Mediators Between Parental Attachment and Gaming Addiction

Social support is a factor that has attracted much interest among researchers seeking to understand what influences video gaming addiction in adolescence (e.g., Moge & Romano, 2020; Yildirim & Zeren, 2021). In general, social support is understood as the feeling of being respected, valued, and cared for by others, especially in times of need (Milienos et al., 2021).

Social support can be divided into two categories: received social support and perceived social support. Received social support focuses on the objective, measurable aspects of support from one’s social network, and emphasizes an objective view of social support. Perceived social support, on the other hand, is based on a person’s subjective feelings and experiences regarding the availability of support and emphasizes a more personal interpretation of this support (Zimet et al., 1988). Essentially, social support received is about the actual support available, whereas social support perceived is about the belief that support is available when needed (Uçur & Dönmez, 2021).

Perceived social support is strongly related to attachment style on both theoretical and empirical levels (Priel & Shamai, 1995). For example, Kobak and Sceery (1988) suggested that individuals with secure attachment are generally open about their distress and are able to seek support from others when needed. In contrast, individuals with ambivalent attachment tend to be overly sensitive to their negative emotions and often focus on them intensely. Avoidantly attached people, on the other hand, have limited awareness of their own emotions and are less likely to seek help or rely on the support of others.

In addition, social support from various sources, including family, friends, and teachers, has been shown to play a crucial role in reducing the likelihood of Internet addiction in adolescents (Cheng et al., 2007; Wu et al., 2016). Longitudinal studies have extended this further by showing that prolonged Internet use specifically for playing online video games in a group of adolescents leads to a decrease in perceived offline social support (Blais et al., 2008).

However, parental attachment and social support may influence video gaming addiction not only directly but also indirectly via other variables, such as maladaptive daydreaming. Maladaptive daydreaming (MD) is defined as “extensive fantasy activity that replaces human interaction and/or interferes with academic, interpersonal or vocational functioning” (Somer, 2002, p. 197). While daydreaming is a common and natural mental experience, MD is a clinical condition in which a person is deeply, often compulsively, immersed in an inner fantasy world, resulting in significant impairment in various essential areas of daily life (Somer et al., 2017). Due to its clinical features resembling those of addictive behavior, MD has been classified by various authors as a form of behavioral addiction (Somer et al., 2016c; Schimmenti et al., 2020).

Empirical evidence suggests that MD is negatively correlated with secure attachment styles and positively correlated with insecure attachment styles, as well as with feelings of shame and dissociative symptoms in community-dwelling adults and self-identified maladaptive daydreamers (Schimmenti et al., 2020). Accordingly, some researchers suggest that MD may act as a form of defensive absorption, that allows individuals to cope with the shame associated with emotional inadequacies in early attachment relationships (Ferrante et al., 2022). Schimmenti et al. (2020) linked MD to feelings of shame and anxious attachment styles, while Ferrante et al. (2022) suggested that MD may also serve as an unhealthy coping mechanism to escape unpleasant emotions and distressing memories related to attachment experiences.

Therefore, in this study, we proposed that MD may be a maladaptive process that, along with low perceived social support, may play a role in the relationship between parental attachment and video gaming addiction. This hypothesis is theoretically supported by Bronfenbrenner’s bioecological systems theory (Bronfenbrenner, 1986; Bronfenbrenner & Morris, 2007), which illustrates how interactions between multiple environmental systems (e.g., parental attachment and social support) and individual characteristics (e.g., MD) influence behavior. Furthermore, MD and video gaming addiction may occur as maladaptive responses to these systemic factors, particularly when secure attachments and social support are lacking.

The rationale for the sequential order of social support preceding maladaptive daydreaming (MD) in the mediation chain is grounded in both theoretical and empirical literature. From an attachment theory perspective, social support is shaped early in development by security or insecurity based on attachment experiences (Mikulincer & Shaver, 2009; Priel & Shamai, 1995). When adolescents have an insecure attachment to their parents, they may have difficulty forming relationships with others who would provide support, resulting in a diminished perception of social support. Decreased social support can lead to emotional distress and increased feelings of social isolation, both of which are associated with an increased likelihood of using inadequate internal maladaptive coping strategies (Ferrante et al., 2022; Schimmenti et al., 2020). In the model presented here, perceived social support is likely to be a more proximal relational form of coping that is directly shaped by parental attachment, and MD is a more individualized psychological form of response that may be employed in the absence of relational or social support. Consequently, this sequence reflects that a lack of social adjustment (low social support) occurs once an intrapsychic (daydreaming) maladaptive delayed response occurs. This model reflects Bronfenbrenner’s theory of ecological systems thinking, which conveys the understanding that one interacts on an individual and contextual level (Bronfenbrenner & Morris, 2007).

### 1.4. Current Study and Hypotheses

The present study is based on Bronfenbrenner’s bioecological systems theory, which assumes that individuals and their environment influence each other over time (Bronfenbrenner, 1986; Bronfenbrenner & Morris, 2007). The study examined the relationship between parental attachment and gaming addiction in a sample of adolescent students. The mediating role of social support and maladaptive daydreaming was also analyzed.

**Figure 1:**
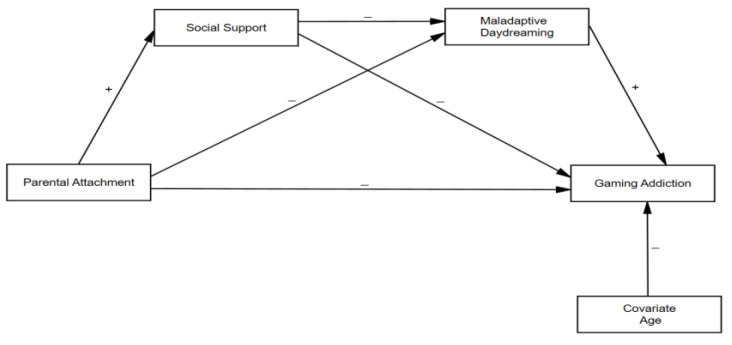
The hypothetical model of serial mediation.

Based on the literature review, we formulated two hypotheses (Figure 1):

**Hypothesis 1** **(H1):** *Parental attachment is negatively related to gaming addiction;*

**Hypothesis 2** **(H2):** *Perceived social support and MD are the serial mediators between parental attachment and gaming addiction.*

## 2. Materials and Methods

### 2.1. Participants

The original sample consisted of 1216 adolescents residing in Italy (male = 461, female = 755) with an average age of 14.81 years (*SD* = 1.78). Missing values in all scales were replaced by the code ‘999’ for identification, and participants with missing values were removed from the dataset. After screening the data for missing values, 318 participants were excluded. The missingness per variable ranged from 0.1% to 2.7%. Little’s MCAR test confirmed systematic missingness (*χ*^2^ (5753) = 7266.26, *p* < 0.001), leading us to compare the imputed dataset (*N* = 1216) and complete dataset (*N* = 898) regression results to assess potential bias (Tabachnick & Fidell, 2019). The results remained consistent across both approaches, with parental attachment (Δ*β* = 0.063), social support (Δ*β* = 0.148), and maladaptive daydreaming (Δ*β* = −0.002). These minor differences suggest that deletion listwise did not substantially alter the results.

The current research included 898 adolescents (*male* = 348, *female* = 550) with an average age of 14.89 years (*SD* = 1.71). These adolescents came from 26 different demographic categories and represented a wide range of regional and national origins, including Italians, Romanians, Moroccans, and Albanians.

### 2.2. Measures

#### 2.2.1. The Inventory of Parent and Peer Attachment (*IPPA*: Armsden & Greenberg, 1987)

The IPPA is a self-report scale designed to measure adolescents’ perceptions of their attachment to their parents and peers. The scale has two parallel versions: 28 items for parents and a version with 25 items for peers. The scale assesses security in attachment relationships based on three dimensions: trust, communication, and alienation. Psychometrically, the IPPA shows good reliability in current research, trust Cronbach’s alpha is 0.82, communication Cronbach’s alpha is 0.81, and alienation Cronbach’s alpha is 0.82.

#### 2.2.2. The Multidimensional Scale of Perceived Social Support (*MSPSS*: Zimet et al., 1988)

The Multidimensional Scale of Perceived Social Support (*MSPSS*) is a widely used instrument for assessing a person’s perceived social support. The MSPSS consists of 12 items and uses a 7-point Likert response format, in which respondents indicate their level of agreement with each statement from *“very strongly disagree” (1) to “very strongly agree” (7)*. The internal consistency of the MSPSS was good in the present study (Cronbach’s alpha = 0.89).

#### 2.2.3. The Maladaptive Daydreaming Scale (*MDS*: Schimmenti et al., 2020)

The 16-item maladaptive daydreaming Scale (Somer et al., 2016a; Italian version by Schimmenti et al., 2020) assesses the degree of maladaptive daydreaming. It comprises two subscales: interference with life (*8 items*) and somatosensory retreat (*8 items*). Participants respond on an 11-point Likert scale ranging from 0% (*never*) to 100% (*extremely frequent*). The MDS-16 has shown excellent psychometric properties in different language versions, including Italian (Schimmenti et al., 2020), English (Somer et al., 2016a), Hebrew (Jopp et al., 2019), Arabic (Abu-Rayya et al., 2020), and Hungarian (Sándor et al., 2020). The internal consistency of the MDS was good in the current study: Cronbach’s alpha = 0.87.

#### 2.2.4. The Game Addiction Scale (*GAS*: Lemmens et al., 2009)

The Game Addiction Scale (*GAS*) consists of seven items that assess the symptoms of addictive video gaming (Lemmens et al., 2009). The GAS was originally developed to assess the symptoms of gaming addiction in adolescents. However, the scale can be used across a wide age range, from 14 to 90 years (Festl et al., 2013). All items are answered on a 5-point scale ranging from *never (1) to very often (5)*, giving a total score of 7 to 35. The internal consistency of the GAS was good in the present study, with a Cronbach’s alpha of 0.80.

### 2.3. Demographic Information Sheet

The demographic data collected included the following: Age, gender, place of residence, and national origin.

### 2.4. Procedure

The research protocol was approved by the University IRB (protocol #914556). In accordance with the ethical code of the Italian Society of Psychology, all participants were informed about the nature and aim of the study.

Individual consent to participate and active parental assent were obtained prior to data collection. Participants were assured of the confidentiality of the data and were informed that participation in the study was voluntary. They could refuse to participate in the study at any time or withdraw from the study if they felt uncomfortable answering a question.

All scales were completed anonymously. All participants were familiarized by a researcher with standard instructions about the requirements of this study. Data were collected in a paper/pencil form. While participants completed the questionnaire, a researcher remained nearby to answer any questions about the items on the questionnaire.

### 2.5. Data Analysis

Data analysis was conducted in SPSS 27. First, descriptive and correlative analyses were performed. Then, the PROCESS macro (Model 6) for SPSS was used to examine the mediating role of social support and maladaptive daydreaming in the relationship between parental attachment and gaming addiction in adolescents. Finally, to further confirm the possible mediating pathways and assess the indirect effects, a bootstrapping procedure was used. The bootstrapping sample size was 5000, drawn from the original sample using random sampling with replacement. This procedure is often used to construct the confidence interval (CI) for indirect effects and no assumptions are made about the shape of the sampling distribution (Hayes, 2022). The indirect effect is considered non-significant if the 95% CI contains the value zero.

To account for potential covariates in the serial mediation model, age and gender were included as covariates, following best practices in mediation analysis (Hayes, 2022). Including gender (either alone or with age) made the second indirect effect (parental attachment → maladaptive daydreaming → gaming addiction) non-significant. However, age kept all indirect effects significant, indicating its key role in maintaining the theoretical coherence of the serial mediation model. Given the minimal collinearity between age and gender (VIF = 1.021), age was retained as the covariate in the final model to ensure accurate mediation estimates.

### 2.6. Causal Inference Limitations

It should be noted that, due to the cross-sectional design of the study, the results show associations and do not allow any conclusions to be drawn about causality. The mediation analysis itself is based on temporal and directional assumptions; therefore, the results are further limited by the lack of longitudinal data. Thus, while the current study provides useful information about the associations between parental attachment, social support, maladaptive daydreaming, and play disorder, no causal conclusions are drawn. Rather, these results should be interpreted as correlative relationships. Future studies with longitudinal designs are, therefore, needed to assess the directionality and causal mechanisms of the associations within the study.

## 3. Results

### 3.1. Correlations and Descriptive Statistics

The results of the correlations and descriptive statistics are shown in Table 1. There was a negative correlation between age, gaming addiction, and parental attachment, suggesting that older adolescents tend to have fewer problems with gaming (*r* = −0.07, *p* < 0.05), and less parental attachment (*r* = −0.13, *p* < 0.001). Of the main variables examined in the model, gaming addiction, social support, maladaptive daydreaming, and parental attachment were significantly correlated with each other. Specifically, gaming addiction was negatively correlated with social support (*r* = −0.26, *p* < 0.001) and parental attachment (*r* = −0.24, *p* < 0.001), and positively associated with maladaptive daydreaming (*r* = 0.29, *p* < 0.001). Social support was negatively correlated with maladaptive daydreaming (*r* = −0.26, *p* < 0.001) and positively associated with parental attachment (*r* = 0.66, *p* < 0.001). Maladaptive daydreaming was negatively associated with parental attachment (*r* = −0.28, *p* < 0.001).

### 3.2. Testing the Serial Mediation

The serial mediation model was analyzed to test the relationship between parental attachment and gaming addiction: mediated by social support and maladaptive daydreaming while controlling for age (see Table 2). Results showed that parental attachment had a statistically significant relationship with social support (*β* = 0.67, *SE* = 0.01, *95% CI* = [0.03, 0.03]) when controlling for age (*β* = 0.06, *SE* = 0.01, *95% CI* = [0.00, 0.05]). Parental attachment (*β* = −0.20, *SE* = 0.03, *95% CI* = [−0.25, −0.10]) and social support (*β* = −0.12, *SE* = 0.78, *95% CI* = [−3.83, −0.76]) were significantly related to maladaptive daydreaming, controlling for age (*β* = −0.07, *SE* = 0.28, *95% CI* = [−1.25, −0.12]). Parental attachment (*β* = −0.08, *SE* = 0.01, *95% CI* = [−0.04, −0.00]), social support (*β* = −0.15, *SE* = 0.24, *95% CI* = [−1.37, −0.39]), and maladaptive daydreaming (*β* = 0.22, *SE* = 0.01, *95% CI* = [0.05, 0.09]) were all significantly associated with gaming addiction, controlling for age (*β* = −0.07, *SE* = 0.09, *95% CI* = [−0.40, −0.04]).

**Figure 2:**
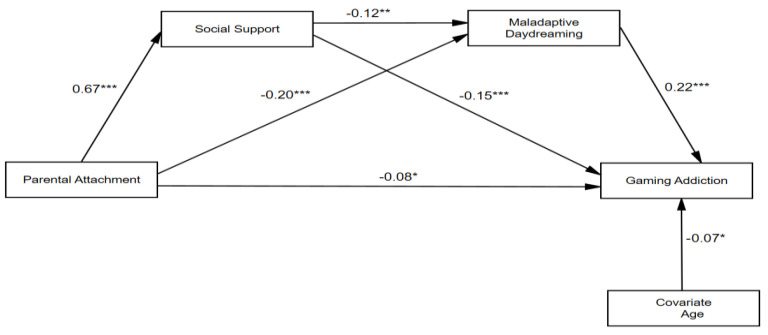
The serial mediation of social support and maladaptive daydreaming between parental attachment and gaming addiction, all results are statistically significant. * *p* < 0.05. ** *p* < 0.01. *** *p* < 0.001.

The serial mediation model tested three mediation pathways (see Figure 2 and Table 3). The indirect effect of parental attachment on gaming addiction through social support was significant (β = −0.1014, 95% CI = [−0.0499, −0.0094]), as was the indirect effect through maladaptive daydreaming (β = −0.0465, 95% CI = [−0.0214, −0.0066]). Finally, the indirect effect of parental attachment on gaming addiction via the serial pathway of social support and maladaptive daydreaming was also significant (β = −0.0192, 95% CI = [−0.0112, −0.0008]). The total indirect effect of parental attachment on gaming addiction was significant (β = −0.1671, 95% CI = [−0.0697, −0.0266]), while the direct effect of parental attachment on gaming addiction was significant (β = −0.0879, 95% CI = [−0.0493, −0.0011]). It confirms that the relationship between parental attachment and gaming addiction is not only direct but also mediated through social support and maladaptive daydreaming.

## 4. Discussion

### 4.1. Correlation Between Parental Attachment and Video Gaming Addiction

The first hypothesis states that parental attachment is negatively related to gaming addiction. The study confirmed a negative correlation between parental attachment and video gaming addiction, which is consistent with previous research (Cui et al., 2018; K. Kim & Kim, 2015), suggesting that secure attachment relationships serve as a protective factor against problem behaviors, including gaming addiction. Adolescents with close parental attachment are more likely to develop emotional security and effective stress coping mechanisms, higher self-esteem and better self-regulation skills, and a reduced need to escape into virtual worlds to find emotional fulfillment or social contact. However, weak parental attachment could lead to unmet emotional needs, that adolescents may seek to satisfy through excessive gaming, a common route for escapism or external validation (D. Kim et al., 2022).

This result is also consistent with recent empirical studies that highlight the crucial role of parental monitoring and mediation in shaping adolescents’ online video game use. For example, in a study involving 452 adolescents, higher levels of parental monitoring were associated with a reduced tendency for adolescents to use video games as a means of coping with negative emotions. Similarly, parental mediation that supports adolescents’ autonomy—such as encouraging independent decision-making and open communication—also showed a protective effect, albeit to a lesser extent (Commodari et al., 2024). Moreover, controlling parental mediation—defined as the use of restrictive rules and enforcement strategies to limit or supervise gaming activities—was found to moderate the relationship between daily game time and gaming disorder (Görgülü & Özer, 2024). In contrast, active parental mediation—characterized by discussing online content with teenage children, co-viewing or co-playing, and guiding critical thinking about digital media—was significantly negatively associated with problematic internet use).

This study underscores that older adolescents tend to have lower gaming addiction problems and lower parental attachment. This result can be explained by several factors. Firstly, adolescents naturally strive for greater independence and emotional distance from their parents as they become older. This reduced parental attachment does not necessarily indicate problematic relationships but is rather a normal developmental shift. Second, older adolescents typically have more mature emotional and cognitive skills that allow them to manage their behavior—including gaming—more effectively. Third, the fact that adolescents at this stage rely on peers rather than their parents as their primary support network may contribute to their ability to balance gaming with other social and academic activities. Bronfenbrenner’s theory provides a useful framework for understanding this relationship, through the concept of “chronosystem” which reflects how relationships, and not just individuals, develop over time (Bronfenbrenner, 1986). As adolescents mature, their relationships with their parents naturally change. Older adolescents generally strive for greater independence, which leads to less parental attachment. This autonomy may also be accompanied by improved self-regulation and the ability to manage their gaming habits more effectively.

### 4.2. The Mediating Role of Social Support and Maladaptive Daydreaming in the Relationship Between Parental Attachment and Video Gaming Addiction

In the statistical model of this study, gaming addiction, social support, MA, and parental attachment were significantly correlated. Initially, gaming addiction was negatively correlated with social support and parental attachment and positively correlated with MD. We have already shown that parental attachment is negatively associated with gaming addiction. We now add that our findings suggest that adolescents with lower levels of social support are more likely to develop gaming addiction, a trend that has also been demonstrated in previous studies (Blais et al., 2008; Cheng et al., 2007; Wu et al., 2016). Gaming can serve as a substitute for meaningful social interaction, especially when real-world relationships are absent or unsatisfying. Online gaming communities can provide a sense of belonging but cannot replace deeper emotional relationships. Gaming addiction and MD share escapist tendencies, as both provide a refuge from real-life challenges or emotional discomfort (D. Kim et al., 2022, in relation to gaming addiction; Somer et al., 2016b, in relation to MD). Adolescents struggling with MD may turn to gaming as an additional way to avoid stressors in the real world.

Second, social support was negatively correlated with MD and positively associated with parental attachment. This may suggest that adolescents with higher levels of social support are less likely to engage in MD. Social relationships may provide emotional outlets and problem-solving resources, reducing the need for mental escape. In addition, adolescents with strong parental attachments are also likely to have robust social support systems, as secure parental attachments often form the foundation for healthy relationships with others (Mikulincer & Shaver, 2009). Positive parents care for role models of empathy, trust, and communication, that adolescents can emulate in their relationships with peers.

Third, MD was negatively related to parental attachment. This finding suggests that adolescents with weak parental attachment are more prone to MD. This behavior is often due to unmet emotional needs or a lack of secure, supportive relationships. Somer and Herscu (2017), for example, have shown that both an unfavorable childhood history and current social isolation are important factors in the development and maintenance of MD. Without a healthy emotional foundation, adolescents may resort to MD as a dysfunctional coping mechanism.

Considering the above associations, this study confirms the three mediating pathways proposed to investigate the second hypothesis, suggesting the mediating role of social support and MD in the relationship between parental attachment and video gaming addiction. First, the study suggests that parental attachment indirectly influences gaming addiction by influencing the level of social support an adolescent receives. Specifically, adolescents with strong parental attachment are more likely to have positive social relationships and robust social support networks. Secure attachment promotes emotional resilience, trust, and interpersonal skills, allowing adolescents to build and maintain meaningful relationships with peers and others (Mikulincer & Shaver, 2009). In addition, a higher level of social support acts as a protective factor against gaming addiction. Socially supported adolescents are less likely to turn to video games to escape loneliness or find a sense of belonging, as their emotional and social needs are met through real-life interactions (Cheng et al., 2007; Wu et al., 2016). The pathway suggests that poor parental attachment can lead to reduced social support, which in turn increases the likelihood of gaming addiction. Conversely, fostering strong parental attachment can improve social support, and thus reduce the risk of problem gaming.

Second, the study highlights the indirect role of MD in linking weak parental attachment and gaming addiction. Adolescents with weak parental attachment are more likely to gamble with MD. Weak emotional attachments to parents can lead to feelings of loneliness, emotional insecurity, or unmet emotional needs (Bowlby & Holmes, 2012). As a result, these adolescents may turn to excessive daydreaming as a coping mechanism or as an escape from emotional distress and unmet psychological needs. This finding suggests that poor parental attachment promotes MD, which, in turn, increases susceptibility to gaming addiction. This link illustrated that emotional dysregulation and escapist behaviors, resulting from weak family attachment are mutually reinforcing. MD, characterized by excessive, immersive fantasizing, often serves as a form of escapism (Nowacki & Pyszkowska, 2024; Somer et al., 2016b). Adolescents who engage in MD may also be drawn to games, which provide a similar outlet to escape the challenges of the real world. Gaming addiction, like MD, can create a fantasy world in which individuals feel a sense of control, fulfillment, or emotional relief.

Third, the study confirmed the relationship between parental attachment, social support, MD, and gaming addiction in a sequential pathway. This suggests a chain of influences in which early attachment influences later psychological and behavioral outcomes via intermediate factors. It shows that early attachment relationships with parents have far-reaching consequences, that extend to certain maladaptive behaviors such as MD and gaming addiction.

The quality of attachment to parents (secure vs. insecure attachment) shapes the way people perceive and interact with the world. People with a secure attachment are more likely to build trusting relationships and regulate their emotions in an adaptive way. In contrast, insecure attachment leads to vulnerabilities such as emotional instability and difficulties in social life (Mikulincer & Shaver, 2009).

This finding suggests that disturbances in parental attachment act as the primary trigger in the chain of subsequent events. Social support bridges the gap between attachment and subsequent events. When parental attachment is weak, it can be difficult for individuals to build supportive relationships, resulting in a diminished social network or perceived availability of support. This highlights a compounding effect: early attachment problems impact social life, creating a reinforcing cycle of emotional distress and social isolation. The lack of adequate social support encourages those affected to rely on internal, fantasy-based coping mechanisms, such as maladaptive daydreaming. This behavior provides a temporary escape from the difficulties of real life but eventually becomes a compulsive habit. The result shows that MD is a central factor in which emotional dissatisfaction and unmet needs turn into escapism and avoidance behavior. Gaming addiction represents the end point of this pathway. The immersive and rewarding nature of gaming serves as an extension of MD and allows individuals to immerse themselves in an alternate reality. The structured feedback loops in games (e.g., achievements, rewards, social interactions) reinforce addiction to games as a form of escapism.

The study emphasizes that gaming addiction is not an isolated problem, but the culmination of unresolved emotional, social, and cognitive vulnerabilities stemming from early life experiences. This finding can also be explained by Bronfenbrenner’s bioecological systems theory, which emphasizes how individual development is shaped by the dynamic interplay between personal characteristics and multiple environmental systems, ranging from immediate contexts such as the family to broader societal influences (Bronfenbrenner, 1986; Bronfenbrenner & Morris, 2007).

## 5. Limitations of the Study and Future Research

Several limitations of this study are worth discussing. First, the data are cross-sectional, meaning that we cannot establish longitudinal correlations to determine how well one variable predicts another over time or to assess the direction of associations between variables. In addition, previous research has highlighted potential biases that can occur when applying mediation analyses in a cross-sectional framework (Maxwell et al., 2011). The mediation pathways were statistically significant, and their effect sizes were relatively small in this study. Such small effect sizes (e.g., β = −0.0192 for serial mediation) are common in psychological research, particularly in complex models involving multiple psychological constructs (Walters, 2018). Even minor statistical effects can be theoretically meaningful when they reflect consistent psychological mechanisms (MacKinnon et al., 2007). To address these issues, future studies could use a longitudinal design to examine these relationships and their direction over time to gain deeper insights into the evolution of associations between variables.

Furthermore, although the sample used in this study was large and diverse, it was a random sample. Therefore, caution should be exercised when attempting to generalize the results to the Italian adolescent population or adolescents from other cultural backgrounds. Future studies with similar objectives would benefit from using more representative samples to improve the generalizability of the results. This approach would allow a more accurate assessment of the transferability of our results to other population groups.

Building on the findings of this study, future research should explore the influence of cultural context in shaping both adolescent behaviors and the interpretation of psychological constructs such as maladaptive daydreaming and parental attachment. As adolescence is experienced differently across sociocultural settings, behaviors like daydreaming or gaming may hold different meanings and implications depending on cultural norms, values, and developmental expectations. For instance, what may be considered maladaptive or excessive in one context could be seen as normative or even adaptive in another. Similarly, perceptions of optimal parental attachment vary across cultures, influenced by differing models of autonomy, emotional expression, and family dynamics (Mesman et al., 2016). We recommend that future studies adopt culturally sensitive frameworks and include cross-cultural comparisons to better understand how these processes function in diverse contexts. This approach may be particularly valuable in refining diagnostic criteria, informing prevention strategies, and tailoring interventions to better support adolescents in varied cultural environments.

Furthermore, this study did not take into account the possible influence of place of residence and national origin, which can have an important impact on gaming behavior. These factors could potentially affect access to gaming opportunities, social norms related to gaming, and the social support system of young people. Future research would benefit from incorporating these factors to examine the role of place of residence and national origin in gaming addiction in different geographic and cultural contexts.

Finally, the variable gender was not considered in our study. Future studies should investigate the role of this variable in the direct and indirect effects between variables.

### Practical Implications

The results of this study emphasize the importance of considering perceived social support and MD in interventions aimed at reducing gaming addiction in adolescents. Schools and community programs can play a critical role by promoting the development of social skills and creating a supportive environment for peers to reduce the sense of social isolation that can contribute to gaming addiction. For example, social skills training programs such as *A Review of Social Skills Programs and Approaches for Autistic Youth* (Moody et al., 2025) can be implemented to help adolescents improve their social interaction abilities, reducing reliance on gaming for emotional fulfillment. Additionally, interventions such as *The Social Competence Program* (Liu et al., 2024) aim to enhance emotional regulation and social interaction in adolescents, further supporting their development outside of gaming.

Psychoeducational workshops for parents and caregivers can also raise awareness of the impact of social support and emotional regulation on adolescent behavior. These workshops could integrate strategies like the *Triple P* (Positive Parenting Program), which equips parents with techniques for managing challenging behaviors and fostering emotional resilience in their children (Sanders, 2023).

Moreover, mental health professionals and educators should identify adolescents who lack social support and focus on teaching them adaptive coping mechanisms. These strategies can help replace maladaptive daydreaming and improve their ability to cope with stress and emotional challenges in a healthier way. Recent studies have shown that the cognitive–clinical approach (Shanbhag & Pothiyil, 2024) and mindfulness (Herscu et al., 2023) can effectively reduce symptoms of MD. Enhancing feelings of safety and self-confidence through such interventions may help to reduce excessive gaming behaviors.

## 6. Conclusions

This study provides valuable insights into the mechanisms linking parental attachment to gaming addiction in adolescents, highlighting the mediating role of perceived social support and MD. The results confirm that secure parental attachment is negatively associated with gaming addiction and that low perceived social support and MD play an important role in this relationship. These findings contribute to a deeper understanding of how interpersonal relationships and psychological processes influence problem gambling behavior.

By highlighting the interplay of these factors, the study emphasizes the need for targeted interventions that address maladaptive coping strategies and strengthen social relationships to reduce the risk of gaming addiction. Future research should examine these relationships longitudinally and in different cultural contexts to further validate and extend these findings.

## Figures and Tables

**Table 1:** Means, standard deviations, and correlations of the variables (N = 898).

Variables	1	2	3	4	5
1. Gaming Addiction	—				
2. Social Support	−0.26 ***	—			
3. Maladaptive Daydreaming	0.29 ***	−0.26 ***	—		
4. Parental Attachment	−0.24 ***	0.66 ***	−0.28 ***	—	
5. Age	−0.07 *	−0.02	−0.04	−0.13 ***	—
*M*	13.49	5.45	39.44	45.64	14.89
*SD*	4.97	0.84	15.37	17.36	1.71

Note: * *p* < 0.05. *** *p* < 0.001.

**Table 2:** Testing the mediation effects of social support and maladaptive daydreaming between Parental attachment and gaming addiction (N = 898).

	Model 1 (Social Support)			Model 2 (Maladaptive Daydreaming)			Model 3 (Gaming Addiction)		
Independent variables	*B*	*SE (B)*	*β*	*B*	*SE (B)*	*β*	*B*	*SE (B)*	*β*
Age	0.03	0.01	0.06 **	−0.68	0.28	−0.07 *	−0.22	0.09	−0.07 *
Parental Attachment	0.03	0.01	0.67 ***	−0.18	0.03	−0.20 ***	−0.02	0.01	−0.08 *
Social Support	-	-	-	−2.30	0.78	−0.12 **	−0.88	0.24	−0.15 ***
Maladaptive Daydreaming	-	-	-	-	-	-	0.07	0.01	0.22 ***
*R* ^2^	0.44			0.09			0.13		
*F*	363.09 ***			21.21 ***			21.55 ***		

Note: *B* = unstandardized coefficient; *SE (B)* = standard error of B; *β* = standardized coefficient; * *p* < 0.05. ** *p* < 0.01. *** *p* < 0.001.

**Table 3:** Standardized and unstandardized direct and indirect effects of parental attachment on gaming addiction. (N = 898).

*Outcome*				*95% CI*	
	*B*	*BootSE*	*β*	*BootLLCI*	*BootULCI*
*Direct effect*					
*Parental Attachment→Gaming Addiction*	*−0.0252*	*0.0123*	*−0.0879*	*−0.0493*	*−0.0011*
*Indirect effect*					
*Parental Attachment→Social Support→Gaming Addiction*	*−0.0290*	*0.0103*	*−0.1014*	*−0.0499*	*−0.0094*
*Parental Attachment→Maladaptive Daydreaming→Gaming Addiction*	*−0.0133*	*0.0038*	*−0.0465*	*−0.0214*	*−0.0066*
*Parental Attachment→Social Support→Maladaptive Daydreaming→Gaming Addiction*	*−0.0055*	*0.0026*	*−0.0192*	*−0.0112*	*−0.0008*
*Total Indirect effect*					
*Parental Attachment→Gaming Addiction*	*−0.0479*	*0.0110*	*−0.1671*	*−0.0697*	*−0.0266*

Note: *B* = unstandardized coefficient; *BootSE* = bootstrapped of standard error; *β* = standardized coefficient; ***BootLLCI***/***BootULCI*** = lower/upper limits of the bootstrapped 95% confidence interval; controlling for age.

## Data Availability

The data that support the findings of this study are available from the corresponding author upon reasonable request.

## Abbreviations

The following abbreviations are used in this manuscript:

CI	Confidence Interval
DSM-5-TR	Diagnostic and Statistical Manual of Mental Disorders, 5th Edition, Text Revision
GAS	Game Addiction Scale
ICD-11	International Classification of Diseases, 11th Revision
IPPA	Inventory of Parent and Peer Attachment
IRB	Institutional Review Board
LD	Linear Dichroism
MD	Maladaptive Daydreaming
MDS	Maladaptive Daydreaming Scale
MSPSS	Multidimensional Scale of Perceived Social Support
SD	Standard Deviation
SE	Standard Error
SPSS	Statistical Package for the Social Sciences
